# Gut‐lung axis and asthma: A historical review on mechanism and future perspective

**DOI:** 10.1002/clt2.12356

**Published:** 2024-04-30

**Authors:** Xiu‐Ling Song, Juan Liang, Shao‐Zhu Lin, Yu‐Wei Xie, Chuang‐Hong Ke, Dang Ao, Jun Lu, Xue‐Mei Chen, Ying‐Zhi He, Xiao‐Hua Liu, Wen Li

**Affiliations:** ^1^ Department of Pediatrics Affiliated Hospital of Guangdong Medical University Zhanjiang China

**Keywords:** asthma, gut microbiota, gut‐lung axis, host immune system, metabolites

## Abstract

**Background:**

Gut microbiota are closely related to the development and regulation of the host immune system by regulating the maturation of immune cells and the resistance to pathogens, which affects the host immunity. Early use of antibiotics disrupts the homeostasis of gut microbiota and increases the risk of asthma. Gut microbiota actively interact with the host immune system via the gut‐lung axis, a bidirectional communication pathway between the gut and lung. The manipulation of gut microbiota through probiotics, helminth therapy, and fecal microbiota transplantation (FMT) to combat asthma has become a hot research topic.

**Body:**

This review mainly describes the current immune pathogenesis of asthma, gut microbiota and the role of the gut‐lung axis in asthma. Moreover, the potential of manipulating the gut microbiota and its metabolites as a treatment strategy for asthma has been discussed.

**Conclusion:**

The gut‐lung axis has a bidirectional effect on asthma. Gut microecology imbalance contributes to asthma through bacterial structural components and metabolites. Asthma, in turn, can also cause intestinal damage through inflammation throughout the body. The manipulation of gut microbiota through probiotics, helminth therapy, and FMT can inform the treatment strategies for asthma by regulating the maturation of immune cells and the resistance to pathogens.

## 1 INTRODUCTION

Asthma is a common chronic airway inflammatory disease characterized by airway hyperresponsiveness (AHR), reversible airflow limitation and airway remodeling. It is mediated by many inflammatory cells, such as eosinophils, mast cells and T lymphocytes,^1^, ^2^ which cause recurrent symptoms involving paroxysmal and reversible attacks of wheezing, shortness of breath, chest tightness, coughing or shortness of breath.^3^, ^4^ The World Health Organization and Global Initiative for Asthma estimated that approximately 300 million people worldwide suffer from asthma, and it is expected to reach 400 million by 2025.^5^, ^6^, ^7^ Especially, an estimated population prevalence of severe asthma at age 10 years is 0.5% and 4.5% among current asthmatic children.^8^ Although most asthmatic children achieve good symptom control by low dose inhaled corticosteroids (ICS) or self‐management, approximately 10% of asthmatic patients are refractory and show resistance to current corticosteroid‐based treatment.^9^, ^10^ Nowadays, the global death rate in childhood asthma still ranges from 0 to 0.7 per 100,000 population.^11^, ^12^ Consequently, further research is urgently needed to shed light on the pathogenesis of refractory asthma and promote the discovery of new treatment strategies for asthma. Recent studies have shown that the gut‐lung axis referring to the crosstalk between gut microbiota and the lung might play an important role in asthma.^13^

Gut microbiota, inherited from the mother to the child through breastfeeding, is a key regulator of gut‐lung axis function and considered to be an important contributor to regulate the homeostasis of the host through physiological, immune and metabolic functions.^14^, ^15^, ^16^ In homeostatic conditions, the healthy gut microbiota can efficiently prevent pathogen infection, reducing the chance of developing inflammation.^17^ On the contrary, early life gut dysbacteriosis may lead to altered immune response and chronic inflammatory respiratory disorders, especially childhood asthma.^18^ Therefore, people are gradually paying attention to the role of gut microbiota and gut‐lung axis in asthma, as well as the mechanism of gut microbiota affecting homeostasis and susceptibility to asthma.^19^ Further understanding of the pathogenesis of asthma, such as the crosstalk between immune factors and gut microbiota or the gut‐lung axis, is expected to provide a new opportunity for the diagnosis and treatment of asthma. This review mainly describes the current immune pathogenesis of asthma, and the role of gut microbiota and gut‐lung axis in asthma. In addition, we are also trying to explore the possibility of manipulating the microbiota, aiming to establish asthma prevention strategies and optimize asthma treatment.

## 2 THE IMMUNOPATHOGENESIS OF ASTHMA

Asthma is a heterogenous disease comprising different phenotypes and endotypes.^20^, ^21^ Based on endotypes, T‐helper(Th)‐2‐high, Th2‐low and the mixed endotypes are described for severe asthma.^22^, ^23^ Th2‐high asthma termed as eosinophilic asthma is associated with innate and adaptive immunity, while Th2‐low asthma associated with non‐eosinophilic asthma is characterized by neutrophilic inflammation or paucicellular inflammation.^24^, ^25^ Studies have confirmed that innate immunity involving macrophages, neutrophils, mast cells and type II innate lymphoid cells (ILC2s) produce a variety of Th2 cytokines, such as interleukin (IL)‐4, IL‐5 and IL‐13, to control the persistence of allergic inflammation.^26^ The ratio tilt of T lymphocyte subsets (Th1/Th2) is the most important pathogenesis of asthma.^27^

Upon allergen stimulation, epithelial‐derived cytokines and ILC2s initiate Th2‐high asthma by driving dendritic cell (DC) activation and phenotypic changes in the airways. On the one hand, airway epithelial cells activate dendritic cells to present antigens, which differentiate naive T cells into effector Th2 cells. Th2 cells amplify type II inflammation through secreting cytokines such as IL‐4, IL‐5 and IL‐13, while Th1 cells mainly mediate type I inflammation by secreting cytokines such as interferon‐γ (IFN‐γ), lymphotoxin (LT)‐α and tumor necrosis factor‐α (TNF‐α) and so on.^28^, ^29^ Th2 cells secrete inflammatory cytokines driving B cells to produce more IgE, which would bind to mast cells to produce a series of inflammatory mediators such as leukotrienes, endothelin, prostaglandin and thromboxane A2, etc. The increased IgE eventually induces rapid onset allergies and chronic airway inflammation.^30^, ^31^, ^32^ On the other hand, airway epithelial cells are involved in polarizing macrophages, DC cells and T cells by producing pro‐inflammatory cytokines such as alarmins IL‐25, IL‐33 and thymic stromal lymphopoietin (TSLP).^33^, ^34^ Besides, alarmins also promote the occurrence of asthma by activating ILC2s. Studies have shown that ILC2 is related to allergic asthma. Under the environment of specific cytokines in patients with asthma, ILC2 facilitates the polarization of Th0 cells into Th2 cells and produces IL‐4.^35^ Increased cytokines such as IL‐5 and IL‐13, rather than IFN‐γ, cause a deviated Th2 cell immune response.^34^, ^36^

**FIGURE 1:**
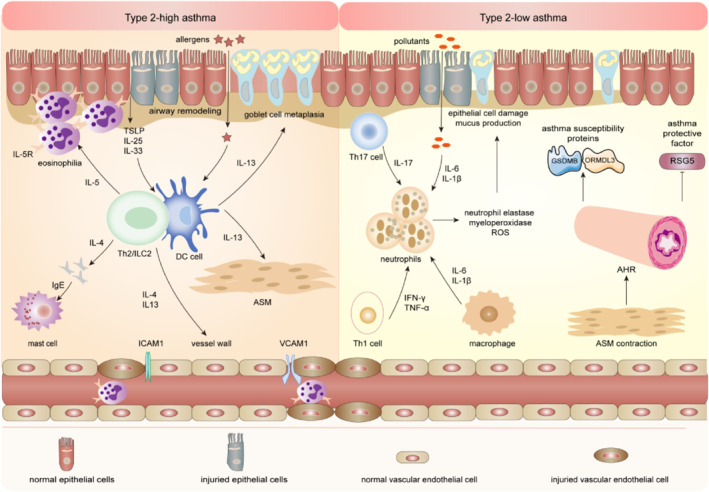
Immunopathogenesis of asthma. Th2‐high asthma: Infected airway epithelial cells release IL‐25, IL‐33 and TSLP, which promote asthma by activating ILC2 and Th2 cells. On the one hand, DC promotes the development of Th0 cells to Th2, resulting in Th1 (decreased secretion)/Th2 (increased secretion) cell dysfunction under IL‐4 induction. On the other hand, ILC2 also polarizes Th0 cells into Th2 cells, which release cytokines such as IL‐4, IL‐5 and IL‐13. IL‐4 acts on mast cells through IgE. IL‐5 acts on eosinophilia. IL‐13 causes goblet cell metaplasia and airway hyperreactivity in ASM. IL‐4, IL‐5, IL‐13 and other cytokines produce IFN‐γ and TNF‐α, causing a balanced skewed Th2 cellular immune response. IgE eventually induce rapid onset allergy and chronic airway inflammation, causing airway remodeling. They can cause vascular endothelial cell injury through intercellular adhesion molecules1 and vascular cell adhesion molecules1. Th2‐low asthma (neutrophilic asthma): Upon pollutants stimulation, the airway epithelium and alveolar macrophages produce pro‐inflammatory cytokines such as IL‐6 and IL‐1β; at the same time, Th17 cells produce IL17, which mediates neutrophil recruitment. Activated neutrophils induce epithelial cell damage and contribute to increased mucus production by releasing factors such as neutrophil elastase, myeloperoxidase or ROS and so on, which result in asthma. Pollutants also contribute to the recruitment of macrophages and Th1 cells to the airways. Th2‐low asthma (paucigranulocytic asthma): Enhanced ASM contraction causes AHR to promote the expression of asthma susceptibility proteins (GSDMB and ORMDL3) and inhibits asthma protective factors like RSG5, both of which together facilitate the development of asthma. AHR, airway hyperresponsiveness; ASM, airway smooth muscle; DC, dendritic cell; IFN‐γ, interferon‐γ; ILC2, type II innate lymphoid cells; ROS, reactive oxygen species; TNF‐α, tumor necrosis factor‐α; TSLP, thymic stromal lymphopoietin.

Th2‐low asthma, simplistically referred to as non‐eosinophilic asthma, encompasses neutrophilic asthma characterized by the activation of Th1 and Th17 cells and paucigranulocytic asthma in which neither eosinophils nor neutrophils are increased.^37^ Upon pollutant stimulation, the airway epithelium and alveolar macrophages produce pro‐inflammatory cytokines such as IL‐6, IL‐1β and so on, at the same time, Th17 cells produce IL17, which mediates neutrophil recruitment.^38^ Activated neutrophils induce epithelial cell damage and contribute to increased mucus production through releasing factors such as neutrophil elastase, myeloperoxidase or reactive oxygen species (ROS) and so on, which result in asthma.^39^ However, paucigranulocytic asthma, which occurs because of AHR caused by enhanced airway smooth muscle contraction, has a low correlation with airway inflammation.^37^ Mechanistically, AHR promotes the expression of asthma susceptibility genes (GSDMB and ORMDL3)^40^, ^41^ and inhibits the expression of critical signaling molecules (RGS5),^42^ both of which together facilitate the development of asthma (Figure 1).

In addition to the pathogenic mechanism of Th1/Th2 imbalance, regulatory T cells (Tregs)‐mediated regulatory mechanism is equally vital in asthma.^43^ Studies have shown an imbalance in the Th17/Tregs ratio in the peripheral blood mononuclear cells of asthmatic patients, with an increase in Th17 and a decrease in Tregs. Treatment with a Th17 inhibitor results in an increase in Tregs and is effective in alleviating asthma.^44^ What's more, oral administration of 14BME20, a *Staphylococcus succinus* strain isolated from soy foods, suppresses airway inflammation by enhancing Tregs responses.^45^

Some immune cells, including ILC2s, ILC3s and Th17 cells, can also produce inflammatory mediators to mediate asthma. For example, ILC2s can transfer from the gut to the lungs, which is involved in lung inflammation and asthma.^46^ Moreover, gut DC senses the gut microbiota to make ILC3s migrate to the lungs, thus producing IL‐22 inflammatory factors that mediate protection against pneumonia.^47^ Th17 cells directly migrate through the lungs and regulate the activities of the pulmonary immune response to mediate asthma.^48^

## 3 THE GUT‐LUNG AXIS AND ASTHMA

### 3.1 Lung microbiota in asthma

Microorganisms can inhabit all surfaces of the human body, including the respiratory tract and gut. The lung microbiota is mainly inhaled through the nasopharynx. Bacteria of *phylum Firmicutes*, *Actinobacteria* and *Bacteroides* are prevalent in healthy lungs. However, *Haemophilus* and *Moraxella* are enriched in asthmatics.^49^ Nasal secretion samples from asthmatic children showed a microbiota dominated by *Moraxella*, a bacterium that induces epithelial damage and inflammatory cytokine expression.^50^ *Haemophilus*, which is enriched in adult asthmatics, induces expression of Th17‐related genes and is associated with worsening asthma^51^ (Table 1). Generally speaking, lung microbiota dysbiosis and subsequently dysregulated microbiota‐related immune response ultimately result in hypersensitivity and hyperreactivity of asthma.

**TABLE 1:** Bacteria related to asthma.

Bacterial Genus	Compartment	Microbiota linked to asthma	Ref
*Clostridium difficile*	Gastrointestinal	*Clostridium difficile* colonization at 1 month associated with asthma at the age of 6 years	52
*Bifidobacterium*	Gastrointestinal	Decrease abundance associated with risk for asthma	53, 54
*Faecalibacterium roseburia*	Gastrointestinal	Decreased abundance in preschool age asthmatic and healthy children at risk for asthma	54, 55
*Rothia, Lachnospira, Veillonella, Faecalibacterium*	Gastrointestinal	Decreased abundance in infants and children at risk for asthma	55
*Lactobacillus rhamnosus GG‐associated fecal products*	Gastrointestinal	Promote expansion of T‐regulatory cells and IL‐10 production, promote tolerance in infants at high risk for asthma	56
*Dolosigranulum, Corynebacterium (C)*	Nasopharyngeal	Prevalence associated with lower risk of viral respiratory infections and asthma in children; *Corynebacterium (C)* negatively associated with eosinophilic lung inflammation in adults	57, 58, 59
*Haemophilus*	Nasopharyngeal respiratory	Increased abundance in early life associated with increased frequency of viral infections and likelihood of developing persistent wheeze	3, 51, 58
*Moraxella*	Nasopharyngeal, respiratory	Increased abundance in early life associated with increased frequency of viral infections and likelihood of developing persistent wheeze	50, 57, 58
*Neisseria*	Respiratory	Increased abundance associated with asthma in adults	57, 58, 59
*Streptococcus clostridium*	Nasopharyngeal, respiratory	Increased abundance in early life associated with increased frequency of viral infections and likelihood of developing persistent wheeze	58, 60
*Veillonella*	Respiratory (R), gastrointestinal (G)	Gastrointestinal (G) decreased abundance in children at risk for asthma	55

### 3.2 The gut microecology imbalance contributes to asthma

Human gut is the most densely colonized part of the body^61^ and inhabits about 10^14^ bacteria that is approximately 10 times the total number of other cells in the human body.^62^, ^63^ It is evident that the imbalance of gut microbiota may cause the host immune homeostasis disorder,^64^ which in turn leads to respiratory diseases such as asthma. There is a link between the abundance of specific gut microbiota and asthma.^65^ A recent study has suggested that breast‐fed infants can effectively prevent childhood asthma and allergic diseases, which may be related to the abundance of specific gut microbiota in breast‐fed infants rather than in formula infants.^66^, ^67^ Moreover, the decrease in the abundance of *bifidobacterium*, *akkermansia*, *faecalibacterium* and increased abundance of *fungi candida* and *rhodotorula* all increase the risk of asthma in children.^68^, ^69^, ^70^ Therefore, we speculate that the changes in the abundance of gut or airway microbiota and the dysbiosis of specific gut microbiota may be potential pathological markers of asthma. Changes in gut microbiota associated with asthma are described in Table 1.

Numerous studies have demonstrated that the bacterial structural components and metabolites of the gut microbiota, such as lipopolysaccharides (LPS) and peptidoglycan as well as multiple mediators, such as short‐chain fatty acids (SCFAs) and desaminotyrosine (DAT), etc., can disrupt the cascade reaction of pulmonary and intestinal immune homeostasis.^71^ On the one hand, gut microbiota can not only provide energy for itself and the host by producing metabolites such as SCFA, but also exert immunomodulatory properties. In addition, SCFA enhances the epithelial barrier function and maintains mucosal immunity. On the other hand, gut microbiota can modify the bile acids synthesized by the liver into secondary bile acids, thus regulating multiple host metabolic processes and immune homeostasis.^72^, ^73^ By contrast, bile acids can affect the growth of bacteria, resulting in changes in the structure of gut microbiota. Moreover, antibiotics and drug exposure can also destroy the stability of gut microbiota, leading to “malnutrition,” that is, dysbiosis of the gut microbiota.^74^ Of course, gut microbiota is also affected by various factors including environment, genetics, diet, and lifestyle. Studies have shown that genetic and environmental factors greatly promote the occurrence of such malnutrition, which leads to the dysbiosis of immune homeostasis and results in disease.^75^

#### 3.2.1 LPS plays a bidirectional regulatory role in asthma

The bacterial components of gut microbiota include LPS and peptidoglycan, which bind to toll‐like receptors (TLRs) or nod‐like receptors (NLRs) as pattern recognition receptors (PRRs) expressed in gut cells to regulate the immune response. Studies have found that LPS is the ligand of PRRs expressed by host antigen‐presenting cells by binding to PRRs (TLRs are the main representative) to trigger a variety of cellular processes that regulate lung immune response.^76^, ^77^ The possible mechanism is that the binding of the LPS‐TLR4 ligand receptor participates in enhancing Th2 and Th1 asthma development.^78^, ^79^ Studies have shown that LPS‐inactivated mice have reduced ability to respond to Th2 inflammation in house dust mite asthma models.^80^ Interestingly, early life LPS exposure was negatively correlated with the incidence of asthma and patterned immune development,^81^, ^82^ which may drive later Th2 asthma. More importantly, it has been observed in animal models that intratracheal perfusion of LPS can lead to changes in the host gut microbiota, suggesting that LPS plays a bidirectional regulatory role in the gut‐lung interaction.^80^, ^83^, ^84^

#### 3.2.2 SCFAs alleviate asthma

SCFAs, the most abundant metabolites of gut microbiota, are considered to be the key mediator of the bidirectional gut‐lung interaction. SCFAs, including butyrate, propionate and acetate derived from the metabolism of dietary fiber by the gut microbiota, are directly proportional to the dietary fiber content.^85^ After they are released into the gut lumen, some SCFAs (especially butyrate) form local immunity in the gut and provide energy for gut cells. SCFAs that are not used in the gastrointestinal tract enter the portal vein and are transported to the liver for metabolism. Access of unmetabolized SCFAs to the peripheral circulation and bone marrow affects immune cell development.^83^ SCFAs may reduce the susceptibility to allergic airway diseases by activating G protein‐coupled receptors (GRP) such as GRP41.^76^, ^86^ Studies have revealed that oral administration of SCFAs to mice can prevent the activation of Th2 cells and the production of cytokines by Th2 cells via weakening DCs, thus protecting mice from the development of asthma.^87^, ^88^ This protection is mediated by changes in hematopoiesis and function of dendritic cells through the gut‐marrow‐lung axis.^89^

Furthermore, butyric acid extracted from dietary fiber has also been shown to enhance blood production of lung anti‐inflammatory macrophage precursors in bone marrow, thereby preventing asthma by controlling immunopathology caused by infiltrating neutrophils.^90^ Recent studies have shown that SCFAs produced by lung microbiota can also mediate the host immune response and cause changes in gut microbiota. This immunomodulatory effect may be played by anti‐inflammatory and anti‐allergic Tregs.

#### 3.2.3 Other metabolites of gut microbiota

DAT and biogenic amines also play an important role in gut‐lung communication. Among them, DAT can regulate the lung response by enhancing the type I interferon response. DAT can protect mice from allergic pneumonia, asthma and influenza virus infection.^91^ In addition, gut microbiota produces metabolites with pro‐ and anti‐inflammatory potential, such as biogenic amines (including histamine)^92^ which have profound effects on asthma through gut‐lung interactions. Both innate and adaptive immune systems can be regulated by histamine. The regulatory action of histamine depends on its binding to four receptors (named H1R‐H4R in the order of discovery).^93^ H3R is mainly expressed in the nervous system and is mostly associated with neuro‐inflammation.^94^ H1R and H4R are thought to be associated with asthma, and their activation can exacerbate asthma.^95^ Histamine has a chemotactic effect on T cells, and H1R‐mediated migration of CD4 T cells into the lung is a key component of the inflammatory response.^96^ Treatment with an H4R antagonist can suppress Th2‐driven asthma and also ameliorates airway dysfunction.^97^ Only H2R agonists may alleviate asthma. H2R antagonist‐treated or H2R‐deficient animals show increased numbers of CD1d^+^ dendritic cells and iNKT cells, which increase inflammatory cells and Th2 cytokines.^95^, ^98^ By contrast, selective H2R agonist treatment reverses these changes and exhibits a protective effect. In addition, histamine is found to promote IL‐10 and inhibit TGF‐α in OVA‐induced asthma in mice, thereby reducing the total number of cells in bronchoalveolar lavage fluid (BALF) and the number of inflammatory factors such as IL‐4, IL‐5, and IL‐13 in lung tissue.^99^ This indicates that the metabolite histamine has a complex immunomodulatory effect on the bidirectional regulation of the gut‐lung interaction. Of course, other gut microbiota metabolites may also participate in immunomodulation.

**FIGURE 2:**
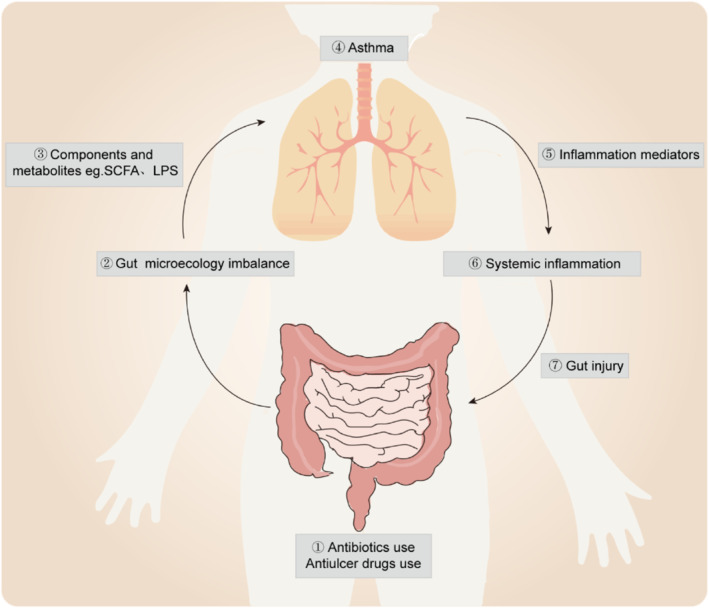
Bidirectional action of the gut‐lung axis. After exposure to antibiotics or drugs, the dysbiosis of gut microbiota leads to gut microecology imbalance. Bacterial structural components and metabolites of the gut microbiota, such as LPS and SCFAs, can regulate the development of asthma. Respiratory disease, in turn, can also cause intestinal damage, creating a vicious circle through inflammation throughout the body. LPS, lipopolysaccharides; SCFAs, short‐chain fatty acids.

As mentioned above, the dysbiosis of gut microbiota is closely associated with asthma (Figure 2). However, studies have also found that the inflammatory response to respiratory infections or allergic inflammatory diseases can lead to gut injury from the respiratory tract to the gut mucosa through the CCL25‐CCR9 axis.^100^, ^101^

### 3.3 The bidirectional action of the gut‐lung axis on asthma

The bidirectional communication pathway between the gut and lung is called the gut‐lung axis.^102^, ^103^ Gut microbiota actively interacts with the host immune system through bacterial structural components and secretion of metabolites, thus regulating the ability of local and systemic immune responses in the gastrointestinal tract, and affecting various distal parts of the host, including the lung, etc. and vice versa.^104^ Pulmonary diseases also affect gut microbiota through lymphocyte migration and inflammatory mediators, leading to gut diseases.^105^

Type II inflammatory asthma involves T helper type 2 cells, ILC2, T follicular helper cells, eosinophils, mast cells, and type II mediators, such as cytokines IL‐4, IL‐5, IL‐13, and prostaglandin D2, etc.^9^, ^106^, ^107^, ^108^ In fact, different types of gut microbiota affect asthma via the gut‐lung axis with different pathological changes. For example, the decreased abundances of gut microbiota such as *lachnospira*, *veillonella*, *faecalibacterium* and *rothia* increase the risk of asthma.^109^, ^110^ *Moraxella catarrhalis* can cause lung neutrophil infiltration, IL‐6 and TNF‐α elevated and moderate levels of CD4T cell‐derived IFN‐γ and lung IL‐17.^69^ By contrast, high doses of ICS treatment in patients with neutrophilic asthma can lead to the transformation of steroid‐sensitive diseases into steroid‐resistant diseases, manifested by relative enrichment of *Haemophilus*.^69^, ^111^ Candida albicans aggravate Th2‐mediated asthma and reduce AHR through the IL‐13‐IL‐33 axis after infection.^69^ On the other hand, clinical studies have found that the levels of inflammatory factors such as C‐reactive protein (CRP) in the peripheral blood of children with asthma are significantly higher than those of non‐asthmatic patients. CRP, the total gut bacterial load and gastrointestinal symptom score (GSRS) are positively related, which indicates that as the levels of peripheral blood inflammatory factors increase, the possibility of gut disorders and gastrointestinal inadaptability in children with asthma will increase.^112^, ^113^ Therefore, this also indicates that bidirectional regulation of the gut‐lung axis and host immunity can mediate gut microbiota dysbiosis and the occurrence of asthma.

There is growing evidence of active and multiple forms of crosstalk between the bi‐directional gut‐lung axis and the host immune system.^114^ This crosstalk is mainly mediated by the following immune cells.

#### 3.3.1 Tregs are regulated by gut microbiota and metabolites

Tregs, a subpopulation of T cells, are important immune cells that mediate host immune responses. There are exact data indicating that gut microbiota in mice mediates the induction of Tregs in the gut and the role of allergen tolerance in the lung. In addition, Tregs also play an important role in host immune diseases such as allergic diseases, but the specific crosstalk between the two is not clear.^115^ This interaction may be caused by blocking the induction of Tregs in the gut, thus leading to Th2‐type inflammation in the lung mucosa. However, more evidence is required to confirm this view. Studies in asthma models have shown that Tregs are regulated by gut microbiota and its metabolite SCFAs. For example, *clostridium* induces Tregs production. SCFAs participate in the differentiation of Tregs to protect asthma.^52^, ^87^ SCFAs increase the expression of transcription factor FOXP3 by inhibiting histone deacetylation, supporting the expansion of Tregs and increasing the production of IL‐10, which in turn interacts with the host immune and participates in asthma.^116^

In addition to the above, there are significant changes in IgA patterns in children with high‐risk asthma. This may be related to the induction of IgA antibodies by Tregs in the gut mucosa and the disordered response of IgA to gut microbiota.^117^

#### 3.3.2 The migration of Th17 cells causes asthma

**FIGURE 3:**
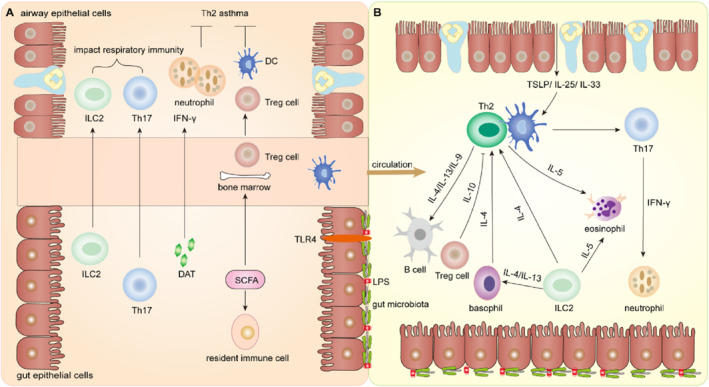
The crosstalk between gut‐lung axis and host immunity. (A) LPS and gut microbiota are important components of gut epithelial cells. TLR4 of gut epithelial cells can recognize the pathogens and activate the immunity. In gut‐lung axis, the structural components and metabolites of gut microbiota such as LPS, SCFAs and DAT, etc. act directly or indirectly on the lung, which initiate immune responses. ILC2 and Th17 cells in the gut migrate to the lung and impact respiratory immunity. DAT produces IFN‐γ and acts on the lung. Neutrophil and DC cells restrain Th2 asthma. SCFAs form local immunity in the gut through resident immune cells, and access to the peripheral circulation and bone marrow, thereby affecting Tregs. (B) The interaction between the gut‐lung axis and host immunity mainly narrates pathological changes of asthma, involving Th2 cells, Th17 cells, other cells, inflammatory factors and mediators. Infected airway epithelial cells release IL‐25, IL‐33 and TSLP, which promote asthma by activating DC and Th2 cells. Th2 cells act on B cells through IL‐4, IL‐13 and IL‐9. Tregs restrain Th2 cells by IL‐10. Basophil and ILC2 cells can act on Th2 cells through IL‐4. ILC2 cells act on basophils through IL‐4 and IL‐13, and on eosinophils through IL‐5. Besides, Th17 cells produce IFN‐γ to mediate neutrophil recruitment. IFN‐γ, interferon‐γ; LPS, lipopolysaccharides; SCFAs, short‐chain fatty acids.

Inflammatory response is a classic case of the interaction between the gut‐lung axis and the host immune system. That is to say, a local immune response originating from the gut tract will lead to different types of host immune cellular infiltrate into the local and distal organs of the lungs then causing pulmonary pathological changes. Nevertheless, asthma can be divided into Th1 or Th2 asthma according to the internal classification. Th2 asthma mainly refers to the asthma with eosinophil infiltration.^118^, ^119^ Th1 asthma is more common in neutrophil infiltration‐based asthma and obesity‐related asthma, which is characterized by Th1 and Th17 cell infiltration and neutrophil infiltration. Studies have found that Th17 cells can directly migrate to the lungs through lymphatic circulation to activate Th2‐type inflammation,^84^ leading to neutrophil inflammation, and thus causing asthma. Therefore, targeting Th17 cells may be a potential therapeutic target for asthma.^120^ Moreover, Th1 asthma is also characterized by the presence of type I interferon, NLRP3 inflammasome activation and so on.^121^, ^122^, ^123^ Conversely, Th17 cell responses induced by pulmonary infection may also lead to gut damage^124^ (Figure 3).

Apart from this, studies have also found that gut microbiota dysbiosis caused by antibiotic exposure in the early life of environmental factors can increase the risk of asthma in adulthood.^125^ The possible pathogenesis of this process is as follows^126^: (1) By increasing the infiltration of inflammatory cells and the production of inflammatory cytokines (IL‐4 and IL‐13), the Th2 response is aggravated. For example, vancomycin treatment of neonates can aggravate the lung inflammation.^87^, ^127^ (2) Reduce the abundance of Tregs in the lungs. Tests have shown that the use of antibiotics almost depletes the bacteria and promotes the overgrowth of lactobacillus.^128^ (3) Amplifying the adaptive immune response of Th1/Th17 in the lung by streptomycin has been shown to exacerbate Th1/Th17 driven inflammatory lung disease and allergic pneumonia.^49^, ^129^ Besides, in large epidemiological studies it has been found that the use of antibiotics and drugs plays an important role in shaping the composition of early gut microbiota.^130^, ^131^ Children receiving a variety of courses of high‐dose and potent antibiotics early in life are predicted to affect the gut microbiota, thereby affecting the host's physiology and immune homeostasis, which may lead to the occurrence of asthma.^132^ The use of antibiotics in the first year of infant life is associated with an increased risk of asthma in young children.^133^, ^134^ Similarly, studies have confirmed that in British Columbia, reduced antibiotic use was significantly associated with reduced asthma risk during the first year of life.^77^, ^135^ Therefore, there is sufficient evidence to support the hypothesis that the use of antibiotics can affect the composition of infant gut microbiota leading to an increase in the incidence of asthma in early childhood.^125^

## 4 MANIPULATE THE GUT MICROBIOTA TO FIGHT ASTHMA

Improving gut microbiota may stimulate its beneficial effects by helping to establish a healthy steady‐state immune balance. This review describes the manipulation of gut microbiota through probiotics, helminth therapy, and fecal microbiota transplantation (FMT) to combat asthma.

### 4.1 Probiotics

Probiotics are oral living microorganisms. Bacteria used for probiotics mainly belong to lactic acid bacteria, actinobacteria, and non‐pathogenic Escherichia coli.^136^ Many studies currently report that probiotics can be used to prevent and treat asthma, especially bifidobacterium and lactobacillus. Studies have found that oral probiotics regulate asthma through many signaling pathways. For example, bifidobacterium can stimulate the balance of Th1/Th2 and up‐regulate the inflammatory factors such as IFN‐γ, IL‐4 and IL‐12 in the lungs to regulate asthma attacks. Oral probiotics can also induce Tregs to reduce the pathological changes of asthma in a mouse model of asthma.^137^, ^138^ Moreover, studies have confirmed that oral probiotic *lactobacillus rhamnosus* GG (LGG) can reduce the expression of matrix metalloprotein 9 (MMP9) in alveolar lavage fluid and serum, inhibit the infiltration of inflammatory cells in lungs and protect asthma.^139^

Apart from the above, asthma children treated with probiotics in clinical studies not only improved their lung function but also reduced the number of asthma attacks. However, the exact mechanism of probiotics' effect on asthma and the regulation of immune response remain unknown.

### 4.2 Helminth therapy

Helminths balance the host immune system to reduce hypersensitivity to allergens by secreting proteins to create an inhibitory environment in the host.^140^ Some studies have confirmed that helminth infection can reduce the morbidity of allergic asthma.^141^ Accordingly, research on helminth therapy provides another clue to the benefits of gut microbiota balance on the respiratory system, and it is also expected to become another clue for the prevention and treatment of asthma. Researchers have discovered that helminths affect the composition of gut microbiota and indirectly affect the lung immune response and prevent asthma attacks.^142^ Studies have shown that mice infected with *Heligmosomoides polygyrus bakeri* will change the composition of gut microbiota by increasing SCFAs and ultimately resulting in reduced inflammation in dust mites‐induced asthma models.^143^

### 4.3 Fecal microbiota transplantation

FMT is a procedure designed to restore the microbiome by transferring feces from a healthy donor into the gut of a recipient.^144^, ^145^ FMT is another way to improve gut microbiota and has been successfully used to improve gut disease, but the potential role of FMT in asthma remains unexplored. FMT current clinical application in asthma is even more limited.^146^ Consequently, further research is needed.

In addition to the methods mentioned above, human trials have shown that by increasing the proportion of fruits, vegetables, fish and probiotic food in the diet, purified metabolites have gradually become promising targets, which can be used as auxiliary intervention strategies for asthma. However, the specific mechanism is not yet clear.

## 5 CONCLUSIONS

Asthma is essentially a heterogeneous inflammatory disease. Different individuals will have different degrees of clinical symptoms, and the age of onset is relatively young, which also seriously endangers people's health. Furthermore, the complex pathogenesis of asthma and the resistance of some children to ICS treatment require people to seek alternative prevention and treatment.

Microbial dysbiosis in lung and gut can be influenced by multiple environmental factors, involving pollution, allergens, use of antibiotics and viruses. Currently, more and more studies have found that the inflammation in asthma seems to be related to the composition of microorganisms and the severity of airway obstruction.^147^ The lung and gut microbiota are considered to be an important part of asthma management. There are significant differences in the abundance of lung microbiota in asthmatics compared with healthy people, which activates inflammatory pathways and contributes to bronchoconstriction and bronchial hyperreactivity.^49^ Many studies have documented that gut microbiota has a regulatory effect on inflammatory diseases. Considering the important role of gut microbiota in inflammatory diseases, it has become a breakthrough for asthma research. By exploring the mechanism of gut microbiota dysbiosis in early life on asthma, researchers hope to intervene and prevent the asthmatic immune response.^56^

It is evident that gut microbiota also plays an important role in distal organs and lungs through immune regulation, which is achieved by bi‐directional gut‐lung axis and host immunity. Therefore, applying the crosstalk between gut microbiota, the gut‐lung axis and immune interaction, the establishment of a specific prevention and treatment model for asthma may be a good prospect for reducing the asthma pandemic.^3^, ^148^ This crosstalk is biologically plausible and ultimately operational, as studies have shown that gut microbiota dysbiosis can be improved and gut microbiota balance is restored through probiotics, helminth therapy, FMT, or purified metabolites to achieve management of asthma prevention and treatment. However, probiotics and FMT have not yet entered clinical routines. Therefore, it is necessary to further explore how to popularize the operation of gut microbiota to benefit asthma patients clinically. Inspiringly, it has been observed through clinical and laboratory studies that asthmatic patients have attempted new therapeutic strategies targeting probiotics, which in most cases have shown encouraging results.

In conclusion, gut microbiota is closely related to asthma, and its dysbiosis increases the risk and severity of asthma. Consequently, the precise role and mechanism behind the changes in structural components and metabolites of gut microbiota and asthma are worthy of further exploration, especially the gut‐lung axis in mediating host immune response and asthma,^149^ even the causal relationship between the gut microbiota and the gut‐lung axis and asthma is still being explored.

## AUTHOR CONTRIBUTIONS

**Xiu‐Ling Song**: Investigation (supporting); visualization (lead); writing – original draft (lead). **Juan Liang**: Investigation (supporting); visualization (lead); writing – original draft (lead). **Shao‐Zhu Lin**: Conceptualization (supporting); formal analysis (supporting); investigation (lead). **Yu‐Wei Xie**: Investigation (supporting); writing – review & editing (supporting). **Chuang‐Hong Ke**: Supervision (supporting). **Dang Ao**: Supervision (supporting). **Jun Lu**: Supervision (supporting). **Xue‐Mei Chen**: Investigation (supporting). **Ying‐Zhi He**: Investigation (supporting). **Xiao‐Hua Liu**: Investigation (supporting). **Wen Li**: Conceptualization (lead); formal analysis (lead); funding acquisition (lead); supervision (lead); project administration (lead); writing – review & editing (lead).

## CONFLICT OF INTEREST STATEMENT

All authors have no conflicts of interest to declare.
